# Development and validation of DSM-5 based diagnostic tool for children with Autism Spectrum Disorder

**DOI:** 10.1371/journal.pone.0213242

**Published:** 2019-03-13

**Authors:** Sheffali Gulati, Jaya Shankar Kaushik, Lokesh Saini, Vishal Sondhi, Priyanka Madaan, N. K. Arora, R. M. Pandey, Prashant Jauhari, Ranjith K. Manokaran, Savita Sapra, Shobha Sharma, Vinod K. Paul, Rajesh Sagar

**Affiliations:** 1 Chief, Child Neurology Division, Faculty in-Charge, Centre of Excellence & Advanced Research on Childhood Neurodevelopmental disorders, Department of Pediatrics, All India Institute of Medical Sciences, Delhi, India; 2 The INCLEN trust International, Phase-I, New Delhi, India; 3 Department of Biostatistics, All India Institute of Medical Sciences, Delhi, India; 4 Department of Pediatrics, All India Institute of Medical Sciences, Delhi, India; 5 Department of Psychiatry, All India Institute of Medical Sciences, Delhi, India; Chinese Academy of Medical Sciences and Peking Union Medical College, CHINA

## Abstract

Diagnostic and Statistical Manual of mental disorder-IV (DSM-IV) TR based INCLEN Diagnostic Tool for Autism Spectrum Disorder (INDT-ASD) is an established instrument for the diagnosis of ASD in Indian subcontinent and low-middle income countries (LMIC). The introduction of DSM-5 necessitated revision of existing INDT-ASD tool to incorporate the DSM-5 related changes. This study was undertaken to develop and validate the DSM-5 based All India Institute of Medical Sciences (AIIMS)-Modified-INDT-ASD Tool. The modifications were done using Delphi method and included: (a) rearrangement of questions from the previous tool; and (b) addition of new questions on sensory symptoms. The modified tool was validated against DSM-5 diagnostic criteria. In addition, receiver operating characteristic (ROC) curves were used to determine the cut-off for total score as compared to Childhood Autism Rating Scale (CARS) score to grade the severity of ASD. Two-hundred-twenty-five children (159 boys, median age = 47months) were enrolled. The modified tool demonstrated sensitivity of 98.4% and specificity of 91.7% to diagnose ASD. A score ≥14 on the tool was suggestive of severe ASD (CARS>36.5) with a sensitivity and specificity of 80% and 80.7% respectively [Area under the curve = 0.89]. AIIMS-Modified-INDT-ASD Tool is a simple and structured instrument based on DSM-5 criteria which can facilitate diagnosis of ASD with acceptable diagnostic accuracy.

## Introduction

Autism spectrum disorders (ASDs) are neurodevelopmental disorders that are characterized by deficits in two core domains: (a) impairments in social interaction and communication; and (b) restricted, repetitive behavior (RRB)[1]. Autism, Asperger’s syndrome and Pervasive Developmental Disorder- Not Otherwise Specified (PDD-NOS) are neurodevelopmental disorders characterized by varying degrees of impairments in social interaction, communication and repetitive behaviors and interests. These disorders lie on a continuum of severity and diagnostic criteria overlap to a great extent[2]. These aforementioned disorders were separately defined in Diagnostic and Statistical Manual of Mental Disorders-Fourth Edition (DSM-IV). However, with DSM-5, a single diagnosis, “Autism Spectrum Disorder”, has replaced the previous subtypes. In addition, in the previous edition of DSM (DSM-IV-Text Revision (TR)) communication and socialization were separate domains; DSM-5 has integrated these to form “deficits in social communication and social interaction” resulting in a two-symptom cluster model[1].

Several diagnostic tools are available to facilitate the diagnosis of ASD. Many of them follow rigid administration standards that may be obtainable only in research setting and some require extensive training[3, 4]. In addition, the expense involved with some of these tools undermines their use in lower-middle-income countries (LMIC). In order to overcome these shortcomings, International Clinical Epidemiology Network validated DSM-IV-TR based INCLEN-Diagnostic-Tool for ASD (INDT-ASD)[5]. The tool demonstrated good psychometric properties and became widely used across Indian subcontinent and other LMIC.

However, with the transition from DSM-IV to DSM-5, and the relative paucity of DSM-5 based diagnostic tools, the INDT-ASD tool required an update. This study was undertaken to: (a) modify existing INDT-ASD tool to incorporate the DSM-5 based criteria and formulate All India Institute of Medical Sciences (AIIMS) Modified INDT-ASD tool; and (b) validate the modified tool against DSM-5 (gold standard).

## Methods

### Participants

The study was conducted at a tertiary care referral hospital of North India between Apr 2015 and Dec 2015. Children aged 1–14 years with “suspected ASD” were enrolled. Suspected ASD was considered when one of following features was present[6]: (a) no babbling or pointing or other gesture by 12 months; (b) no single words by 16 months; (c) no 2-word spontaneous (not echolalic) phrases by 24 months; or (d) loss of language or social skills at any age. As per American Academy of Neurology and Child Neurology Society, such children require further developmental assessment and screening for ASD. The study was approved by the Institutional Ethical Committee, All India Institute of Medical Sciences, New Delhi.

### Outcome measures

This study was undertaken to develop and validate the AIIMS Modified INDT-ASD Tool, against DSM-5 based expert diagnosis, in children aged 1–14 years. The primary outcome was to assess the psychometric property of the aforementioned tool (accuracy and correlation with CARS score). Secondary outcome was development of severity scoring for ASD in this tool.

### Development of AIIMS modified INDT ASD tool for ASD

A team of Paediatric neurologists, clinical psychologists and psychiatrist reviewed clinical criteria for ASD as presented in DSM-5, ICD-10, DSM-IV TR, CARS and INDT-ASD tool. Subsequently, questions from INDT-ASD tool (12 items– 4 each in social interaction, social communication, and RRBs) were selected and rearranged into seven items (three for social interaction/ communication and four for RRBs). Additional questions for sensory symptoms were pooled and reviewed by a team of experts using modified Delphi Technique. These pooled questions were rank-ordered and further reduced using endorsement rate approach. In this process, 5 questions from INDT-ASD tool were dropped and 4 new questions (sensory symptoms) were added. Key differences in diagnostic criteria for autistic disorder and ASD using DSM-IV and DSM-5 based tools are illustrated in Table 1. The AIIMS modified INDT-ASD tool has been illustrated as supporting information (S1 File).

**Table 1:** Key differences in diagnostic criteria for autistic disorder (DSM IV based INDT ASD tool) and ASD DSM-5 based AIIMS modified INDT ASD tool.

Parameter	DSM-IV based INDT ASD tool	DSM-5 based AIIMS modified INDT ASD tool
Social interaction	4 Subdomain (A1a, A1b, A1c, A1d)	3 Subdomain (A1a, A1b, A1c)
Social communication	4 Subdomain (A2a, A2b, A2c, A2d)	
Restrictive and repetitive behaviour	4 Subdomain (A3a, A3b, A3c, A3d)	4 Subdomain (A2a, A2b, A2c, A2d)
Sensory symptoms	Absent	Present (1 item) out of 4 items in restrictive repetitive behaviour (A2d)
Impairment of daily functional activity	Absent	Present (1 item: Section B, Question 4)
Total number of items	12	9
Diagnosis of ASD	6 out of 12 criteria for diagnosis of autistic disorder	7 out of 9 criteria needed for diagnosis of ASD

DSM: Diagnostic statistical manual; INDT: International Clinical Epidemiology Network tool for autism spectrum disorder; ASD: Autism spectrum disorder; AIIMS: All India Institute of Medical Sciences

### Components of AIIMS modified INDT ASD tool

The modified tool has two sections (Section A and Section B). Section A has 28 questions for 7 items (3 items for social interaction/communication and 4 items for RRBs); representing domains of DSM-5 criteria for ASD diagnosis. Section A has 2 subsections: Subsection A1 has three subdomains namely, deficits in social-emotional reciprocity (A1a;8 questions), non-verbal communication (A1b;4 questions) and deficits in developing and maintaining relationships (A1c; 3 questions) and subsection A2 has 4 subdomains namely- Stereotyped movements or speech (A2a;7 questions), Fixed routines (A2b;1 question), Fixed interests (A2c;1 question) and Sensory symptoms (A2d;4 questions).

Response to each question is marked as “yes”, “no” or “unsure”. Response of unsure is marked only when both parents and investigator (based on observation) are unsure of the response. Investigator assessment relies upon interview of primary caregivers and direct observation of child involved in spontaneous play activity. For any discrepancy in parental response and investigator’s assessment, it is indicated for each question whether parental response or assessor’s observation should take precedence. Based on question and indication in the tool, the response of either “yes” or “no” might be “abnormal”. Number of “abnormal” responses are calculated as “total score” for each patient. Hence a child may score anywhere between zero to 28.

Section B has 9 questions for analysis of items in section A. All three subsections of section A1 (A1a, A1b, A1c) along with at least 2 out of 4 subsections of section A2 (A2a, A2b, A2c, A2d) must be “abnormal” to qualify for the diagnosis of ASD. In addition, Section B has two mandatory items- onset in early developmental period and impairment in daily functioning that is a prerequisite for diagnosis of ASD.

### Pilot testing

All investigators participating in the trial underwent training for application of the tool. The training, that took four hours, was performed using lectures and by practical application of tool on patients. Pilot testing was done in 20 subjects (aged 1–14 years) who were already diagnosed with ASD (based on DSM-5 criteria) in the preceding three months. Internal consistency of items as a whole construct was good with Cronbach alpha 0.92 (individual Cronbach alpha ranging from 0.81–0.89). The inter-rater reliability of tool was very good, and kappa was observed to be 0.95 (95% confidence interval (CI) = 0.85, 1.00).

### Study enrollment

**Fig 1:**
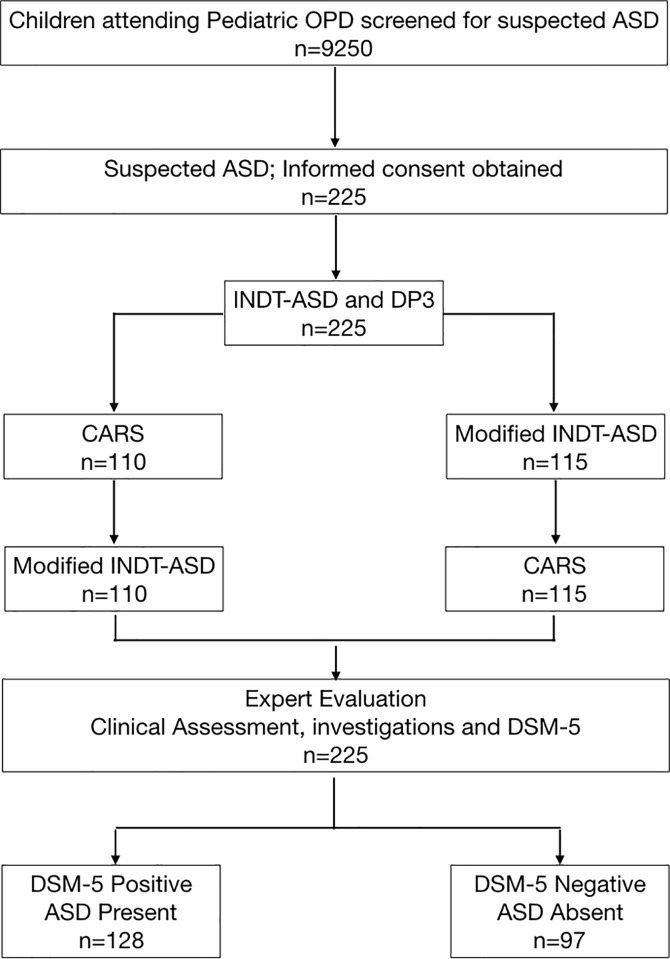
Flow of patients.

Eligible children attending pediatric outpatient unit were screened consecutively for presence of “suspected ASD” by questioning caregivers for presence of any of the four screening criteria. Those who screened positive were invited to participate in the study. Written informed consent was obtained from parent/ guardian of every child participating in the study. INDT-ASD tool and Developmental profile-3 (DP-3, to assess developmental quotient(DQ)) [7] were administered by Investigator 1. Subsequently, CARS and AIIMS modified INDT-ASD tool were administered by Investigators 2 and 3. The sequence of administration was CARS (Investigator 2) followed by AIIMS modified INDT-ASD tool (Investigator 3) in one group and in another group, AIIMS modified INDT-ASD tool (Investigator 2) followed by CARS (Investigator 3). This sequence was adopted to minimize rating bias. Study subjects were finally evaluated independently by a team of experts (Gold standard/Investigator 4). Each evaluator was blinded to original diagnosis and assessment results of other evaluator; their evaluations were separately sealed in opaque envelops immediately after assessment. Study flow is illustrated in Fig 1.

Gold standard assessment was performed by a team consisting of three members: one paediatric neurologist, one clinical psychologist and one child psychiatrist. Expert review was based on history and observation of the child for possible fulfilment of DSM-5 criteria and final diagnosis was categorized as presence or absence of ASD. Patient’s treatment and management plan were guided by assessment done by the team of experts. A consensus of diagnosis was reached among team members after a round table discussion. If there was a discrepancy on more than two clinical features, the study participant was reassessed next day by a different set of team members. In case of persistent discordance, all members of gold standard assessment team reached a consensus diagnosis by discussion.

### Statistical analysis

Sensitivity, specificity, positive and negative predictive value of AIIMS modified INDT-ASD tool was calculated by comparison with DSM-5 based expert diagnosis. Correlation between severity as per this tool and by CARS was assessed using Pearson correlation. Receiver Operating Characteristics (ROC) curve was used to determine the cut off score for diagnosis and for cut off score (as compared to CARS score) to diagnose severe ASD. STATA version 13.0 was used for statistical analysis.

## Results

Two-hundred-twenty-five children were enrolled (159 (70.7%) males). None refused consent to participate. The median (IQR) age of study cohort was 47 (36, 63.5) months. The baseline characteristics of cohort have been illustrated in Table 2. One-hundred-twenty-eight (56.9%) of 225 enrolled children were diagnosed as ASD based on gold standard assessment. Overall, nearly 51% children had development quotient (DQ) ≤50. The proportion of children with DQ≤50 was significantly higher among children with ASD (60.2%) than those without ASD (39.2%, p = 0.002). Thirteen (5.7%) children (nine males) were in age group of 1–2 years. The median age of the ≤2years subgroup was 18months (15-24months). One of the thirteen children, in the age group ≤2years, was ASD positive by DSM-5 and he also tested positive by the tool.

**Table 2:** Baseline data.

Variable	Complete Cohort n = 225	ASD +ve by DSM n = 128	ASD–ve by DSM n = 97
**Age (months)**			
Mean ± SD	54.59±29.14	58.58±31.15	49.33±5.47
Median	47	49	44
IQR	36–63.50	37–74	32.50–53.50
Range	15–180	22–180	15–146
**Gender, N(%)**			
Males	159 (70.7%)	99 (77.3%)	60 (61.9%)
**Development Quotient, N(%)**			
≤50	115 (51.1%)	77 (60.2%)	38 (39.2%)
51–60	64 (28.4%)	43 (33.6%)	21 (21.6%)
61–70	33 (14.7%)	5 (3.9%)	28 (28.9%)
71–80	8 (3.6%)	2 (1.6%)	6 (6.2%)
81–90	2 (0.9%)	0	2 (2.1%)
>90	3 (1.3%)	1 (0.8%)	2 (2.1%)
**CARS Score, N(%)**			
No autism (<30)	106 (47.1%)	11 (8.6%)	95 (97.9%)
Mild to Moderate (30–36.5)	39 (17.3%)	37 (28.9%)	2 (2.1%)
Severe (≥37)	80 (35.6%)	80 (62.5%)	0

Diagnostic performance of AIIMS modified INDT-ASD tool against gold standard DSM-5 based expert diagnosis revealed sensitivity (95% CI) and specificity (95% CI) of 98.4% (94.5%-99.8%) and 91.7% (84.4%-96.4%), respectively (Table 3). Pearson correlation between diagnosis based on CARS and the modified tool for ASD was 0.76 (p<0.01) (Table 4).

**Table 3:** AIIMS modified INDT-ASD tool validation statistics as compared to gold standard (DSM-5).

	Gold standard (DSM-5 based expert diagnosis)		Total cases
	ASD present (n = 128)	ASD absent (n = 97)	
AIIMS modified INDT ASD tool: ASD **present**	126	8	134
AIIMS modified INDT ASD tool: ASD **absent**	2	89	91
	128	97	225
a. Sensitivity: 98.44% [94.47% to 99.81%] b. Specificity: 91.75% (84.39% to 96.37%) c. Positive Predictive Value: 94.03% (88.58% to 97.39%) d. Negative Predictive Value: 97.80% (92.29% to 99.73%)			

AIIMS: All India Institute of Medical Sciences; INDT: International Clinical Epidemiology Network tool for autism spectrum disorder; ASD: Autism spectrum disorder; DSM: Diagnostic statistical manual of mental disorders

**Table 4:** Diagnostic performance of AIIMS modified INDT ASD tool across Childhood Autism Rating Scale (CARS) severity.

CARS Severity	Tool Positive	Tool Negative
Non autistic (CARS<30) (n = 106)	11 (10.4%)	95 (89.6%)
Mild to moderate (CARS = 30–36.5) (n = 39)	37 (94.9%)	2 (5.1%)
Severe (CARS>36.5) (n = 80)	80 (100%)	0

AIIMS: All India Institute of Medical Sciences; INDT: International Clinical Epidemiology Network tool for autism spectrum disorder; ASD: Autism spectrum disorder; DSM: Diagnostic statistical manual of mental disorders

The modified tool was false positive in 8 of 97 cases (8.2%). The final diagnosis of false positive cases included- Intellectual Disability (ID- 6) and Social Communication Disorder (SCD- 2). Similarly, numbers of cases that were falsely diagnosed as ‘no ASD’ by tool were two out of 128 (1.55). Both cases were down by one criterion among stringent 3 out 3 criteria in section A1.

**Fig 2:**
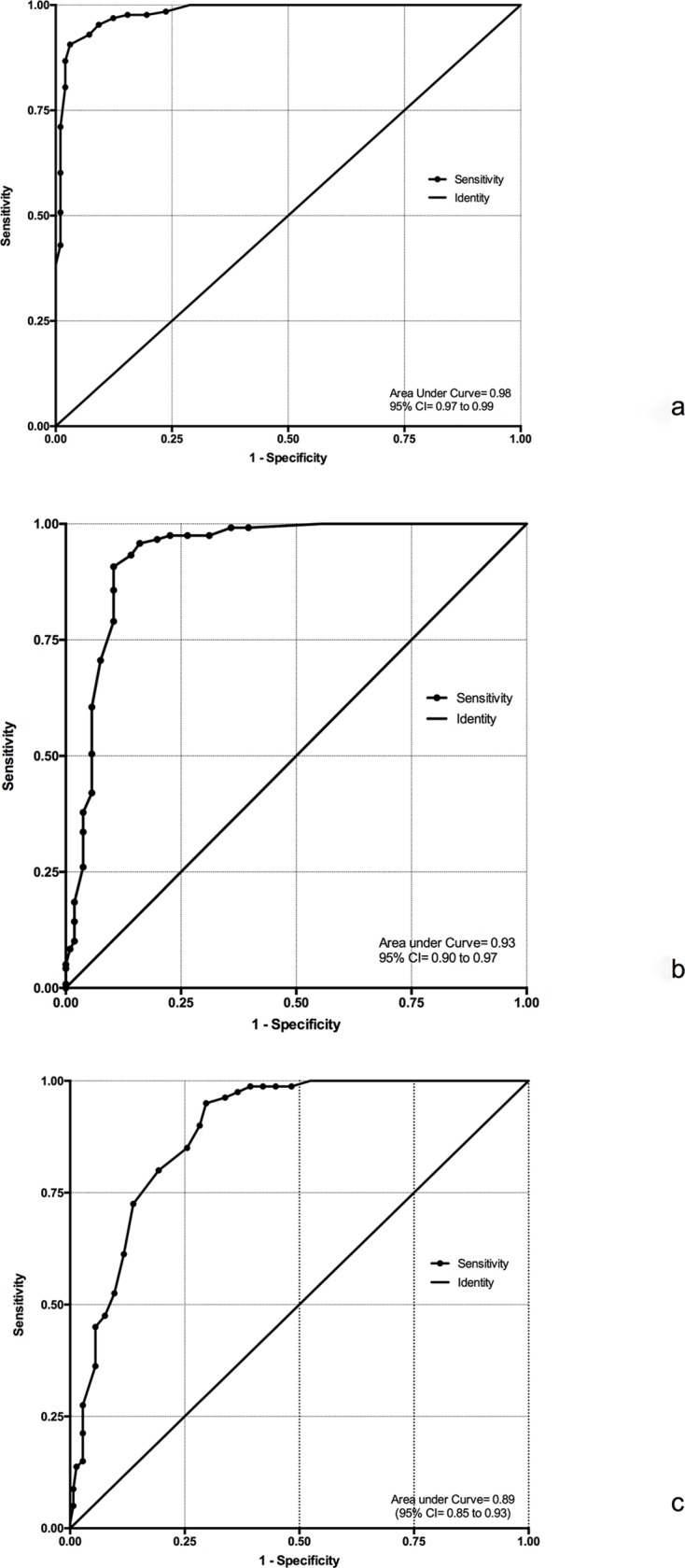
Receiver operative characteristics (ROC) curves revealing: (2a) ROC demonstrates that at AIIMS Modified INDT-ASD score of >10, ASD can be diagnosed with sensitivity and specificity of 92.97% and 92.98% respectively (Area under curve (AUC) = 0.98); (2b) ROC demonstrates that with AIIMS Modified INDT-ASD score of >11, “moderate ASD” (CARS score of 34–36.5) can be diagnosed with sensitivity and specificity of 90.76% and 89.62% respectively (AUC = 0.93); and (2c) ROC demonstrates that with AIIMS Modified INDT-ASD score of >14, “severe ASD” (CARS score>36.5) can be diagnosed with sensitivity and specificity were determined to be 80% and 80.69% respectively (AUC = 0.89).

A score of ≥10 on this tool diagnosed ASD with sensitivity and specificity of 92.97% and 92.98% respectively (AUC = 0.98). The cut-off score to diagnose moderate ASD (CARS score 34 to 36.5) was ≥11 with sensitivity and specificity of 90.76% and 89.62% respectively (AUC = 0.93). Similarly, the cut-off score for diagnosing severe ASD was ≥14, which corresponded to CARS score of >36.5. At this cut-off, the sensitivity and specificity were determined to be 80% and 80.69% respectively (AUC = 0.89) (Fig 2).

## Discussion

We demonstrate that DSM-5 based AIIMS modified INDT-ASD tool has good psychometric properties for diagnosis of ASD. In our cohort, the tool demonstrated a sensitivity and specificity of 98.4% (95% CI = 94.5%-99.8%) and 91.7% (95% CI = 84.4%-96.4%), respectively. The modified tool had false positivity of 8.2%, while false negative rate was 1.55%. These properties are also supported by its correlation with severity on CARS; with a score of ≥14 on this tool predicts severe ASD with sensitivity and specificity of almost 80% each.

DSM-5, currently constitutes the standard criteria available for ASD diagnosis. Various tools that are available for diagnosis of ASD are DSM-IV or ICD-10 based. Currently, ‘gold standard’ diagnosis of ASD is a protracted and time-consuming process that requires a qualified multi-disciplinary team to assess behavioral and parent-report information. Considering the relative inaccessibility of LMICs to gold standard for ASD diagnosis and also the expense involved with some of the tools, it is essential to freely adapt, translate and validate diagnostic tools as needed for use in diverse cultures and settings. INDT-ASD tool was an attempt in this direction and it facilitated ASD diagnosis by using appropriateness criteria developed for Indian context. The INDT-ASD tool had sensitivity of 98% and specificity of 95.1% against DSM-IV diagnosis of autism[5]. With the transition from DSM-IV to DSM-5, we modified the existing INDT-ASD tool to incorporate the DSM-5 based questions. The AIIMS modified INDT-ASD tool, thus generated, has also shown good diagnostic accuracy. To the best of our knowledge, this is the first DSM-5 based diagnostic tool for ASD in children. Considering the incorporation of DSM-5 criteria for diagnosis, expansion of age range to 1–14 years and comparable psychometric properties, present tool can replace the previous tool for ASD diagnosis.

As per the evidence, ADI-R and ADOS are considered the best tools for ASD diagnosis with correct classification rates (as per DSM-IV) of up to 80.8%[8]. Few studies using combined ADOS and ADI-R ratings show that this combination has stood the test of time even after transition of DSM-IV to DSM-5[9, 10]. In a recent study, Developmental Diagnostic Dimensional Interview-short version (3Di-sv) proved to be a solid basis for a diagnostic tool to build upon (based on DSM-5) with some modifications[11]. Though we do not have a direct comparison between our tool and any of the aforementioned tools, but nonetheless we demonstrate sensitivity and specificity in excess of 90% for the AIIMS modified INDT-ASD tool.

ASD as a diagnosis has immense phenotypic heterogeneity in terms of symptom severity, verbal and non-verbal IQ, and social attention[12, 13]. On one end of this spectrum are the children with impaired intellectual capabilities and at the other end are children with autism with average or above average intellectual abilities (high functioning autism)[12]. The elimination of sub-diagnosis in DSM-5 have led to concerns that DSM-5 criteria may underdiagnose ASD and especially impact the Asperger and Pervasive Developmental Disorder (PDD) end of spectrum. Studies have however, indicated that most individuals with a prior DSM-IV PDD meet DSM-5 diagnostic criteria for ASD and SCD[14, 15].

Screening criteria adopted in the present study are one of the objective criteria available in literature, for surveillance of children, to find those who need screening for ASD. Though these screening criteria are dominated by language delay, these may be useful for ASD screening in LMICs where standard screening tools are not available. Extrapolating these criteria for screening children (for broad age-group: 1–14 years) might raise a concern of losing out on children with good verbal abilities and those with high functioning ASD. This concern is similar to that of applying DSM-5 criteria on those diagnosed with ASD based on DSM-IV. This could probably explain the relatively comparable diagnostic performance of DSM-5 based new tool (sensitivity = 97%) with DSM-IV based INDT-ASD tool (sensitivity = 98%). This might also be contributory for majority of enrolled children in present study having a DQ <70.

In the present study, it was observed that six children with ID and two with SCD (median age of 49 months) were labelled as ASD by the tool; thereby, raising a concern of misdiagnosing ID and SCD as ASD. This tool, akin to DSM-5 criteria, renders provision for co-existing diagnosis of ID with ASD and liberty to mark that features of ASD can be explained by ID. Therefore, the final diagnosis might not suffer when test for cognitive abilities are used in conjunction with the tool.

Existing tools for diagnosis of ASD are based on ICD 10 and DSM-IV[5, 16, 17]. Current study developed a well-structured, user-friendly, physician-administered DSM-5 based tool for diagnosis of ASD. This tool is easy to administer and requires minimal training. Good internal consistency of the tool (Cronbach alpha 0.92) demonstrates that symptom cluster of the modified tool was homogenous.

The biggest strength of the study is development of an updated DSM-5 based diagnostic tool to facilitate diagnosis of ASD especially in LMIC which may have limited access to other commercially available tools. In addition, a robust study design, and adequate sample size add to strength of this study. The present study has a few limitations. Firstly, this study lacks concurrent comparison of AIIMS Modified INDT-ASD Tool with ADOS and ADI-R. However, this was beyond the scope of this study. We primarily aimed to modify the existing tool and compare it with DSM-5 and not with other tools. Secondly, there was limited enrollment in the age group of 1 to 2 years. However, we feel that diagnosis of ASD is still evolving in children less than 2 years of age. We still have to identify early markers before we can conclusively diagnose ASD in this population. Thirdly, the applicability in other LMICs needs further evaluation. And finally, its utility as a diagnostic tool for ASD among children suspected with autism might raise a concern considering specificity of 91.7% (84.4%-96.4%) and false positivity rate of 8.2%. However, administration of other diagnostic instruments for cognitive assessment could avert this concern. Hence, the present tool can become a part of the comprehensive assessment of ASD that consists of an assessment of symptom cluster, cognition, language, and speech.

To conclude, DSM-5 based AIIMS modified INDT-ASD tool has good psychometric properties for diagnosis and for severity rating of ASD among children aged 1–14 years. Hence, the present tool offers simple, physician-administered, diagnostic and severity instrument for ASD among children with “suspected autism”.

## Supporting information

S1 File AIIMS modified INDT-ASD tool for diagnosis of autism spectrum disorder. (PDF)

S2 File Deidentified dataset. (XLSX)
