# Supplementary material for: Development and validation of DSM-5 based diagnostic tool for children with Autism Spectrum Disorder
Source: PLoS One. 2019 Mar 13;14(3):e0213242. doi: 10.1371/journal.pone.0213242 (PMC6415840; doi:10.1371/journal.pone.0213242)
Supplement: S1 File — (PDF) [file pone.0213242.s001.pdf]

AIIMS Modified INDT-ASD Diagnostic Tool for Autism Spectrum Disorder

Name of the Child: ______________________________

Date of Birth: DD/MM/YYYY Age: _ _ years _ _ months

Sex:

Complete Address:

Phone number:

Date of Assessment: DD/MM/YYYY

Name of the Assessor:

INSTRUCTIONS FOR EVALUATION

Primary caregiver must be present with the child

- These behaviors are to be assessed in the context of children of **same age**
- Explain to parents that the answers should be based on the child's **behavior most of the time**
- Follow the age directions given along with the question. For questions where no age cut-off is given, they should be asked for all children i.e. all ages (1-14 years)
- Ask the questions **verbatim**

Question can be **repeated** if the respondent can not understand

Still, if the respondent cannot understand, give **example** for the particular behavior;

No further elaboration is allowed

- The questionnaire should be **supplemented by observations** for the suggestive behavior in the child **throughout** the assessment.
- Observe the behavior of child during the entire interview to confirm the presence or absence of a particular behavior (First ask, then observe if observation is discrepant, then re ask the question and re-check the observation)
- When there is discrepancy between parental response and your observation, * indicates whether parent report or observation should take precedence, and marked accordingly

- When the parent's response is "unsure" your observation of the particular behavior will be given weightage even asterisk (*) is on parental response. In case you are also unable to observe the behavior, and then only mark the response as "Unsure".
- Some criteria have multiple questions. **While scoring,** consider the criteria fulfilled even if response to **any one** of the questions is abnormal. For example, the criterion A1a is considered fulfilled if any one of i, ii, iii, or iv is abnormal in the child

AIIMS Modified INDT-ASD Diagnostic Evaluation for ASD

Section	Ask	Observe	Yes	No	Unsure
A1a<br>Social emotional reciprocity	i) * ***For children aged less than 4 years:*** Does/did your child **ever** point with his/her index finger to bring your attention to show the things that interest him/her ? *E.g. kite, plane flying in the sky, cow/dog on the road etc.* ***For children aged 4 years or more:*** Does your child **usually** bring things to show you on his/her own he/she has made painted or new toy/gift?	Observe how the child draws attention toward a toy/object of interest; Look for coordinated pointing		○	
	ii) * ***For children aged 4 years or more, and are able to speak***: Does your child talk to you about things he/she likes or has achieved without being asked about them?			○	
	iii) * Does your child **usually** prefer to play alone **and** gets irritated/moves away when his/her sibs or other kids try to play with him/her?	Quality of play activity in a group of children or with siblings	○		

	iv) * Does your child play **games involving turn taking or rule based** with other children **properly**? *E.g. Cricket, Hide and seek/I-spy, Ludo,Stapoo, Ring-a- ring roses etc.*	Quality of child's involvement in rule-based games or games involving taking turns		◯	
	v) * Does your child **usually** share his/her happiness with you or come to you for comfort when hurt or upset?	Sharing happiness or distress with the parents		◯	
	vi) * ***For children aged 4 years or more:***<br>Does your child **usually** share your<br>happiness or try to comfort you when you are upset / sad?	Sharing of parent's happiness or distress by the child		◯	
	vii)* Does your child **initiate** a conversation with you?	Quality of child's conversation with parents or yourself		◯	
	viii)* ***For children aged 4 years or more:*** Can you have conversation with your child during which he/she not only answers your questions, but also **adds** something new **to continue** the conversation?	Quality of child's conversation with parents or yourself		◯	

Section A1b<br><br>Non verbal communication	i) * ***For children aged less than 4 years:***<br><br>Does your child **usually** enjoy being taken in the lap or hugged?***For children aged 4 years or more****:* When your child was a baby/toddler, did he/she enjoy being taken in the lap or hugged?	In children below 4 years age: Response to being touched and cuddled by parent: enjoys/tolerates/squirms/ stiffens/ gets upset/ Indifferent		○	
	ii) Does your child **usually** make eye contact with you or other people?<br><br>*E.g. While playing, asking for things, talking to you.*	* Quality of eye contact		○	
	iii) * Does your child **usually** use various gestures appropriately during social interactions?<br><br>*E.g. Namaste, Salaam,* ***waving*** *bye-bye, hello, touching feet etc.* (At least sometimes spontaneously) *(use appropriate example as required)*	Use of these gestures in response to your greeting and while departing		○	
	iv) Does your child **usually** show<br>appropriate facial expressions according to the situation?<br>*E.g. being happy, sad, afraid etc.*	*Appropriateness of facial expressions while interacting with parents, with you (stranger), while playing, when given toy/favorite food or when scolded.		○	

Section A1c<br>Relationships	i) * Does your child **usually** enjoy the company of other children?	Child's interaction with other children			
	ii) * ***For children aged 4 years or more:*** Does your child have friends of his/her age (In school and neighbor-hood) with whom he/she loves to chat, share food or play together?	Quality of child's interaction with other children of his/her age			
	iii) * ***For children aged 4 years or more:***<br>Does your child play **mostly** with children who are much older or much younger than him/her?	Quality of child's interaction with other children			
Section A2a<br>Stereotyped movement or speech	i) Does your child **usually** repeat words or phrases regardless of meaning (in part or whole) that he/she has heard?<br>*E.g. If you say 'toffee' he will also say 'toffee' if you say 'come' he will also say 'come' and if you ask "what is your name", he/she also says "what is your name".*	* Immediate echolalia (words or phrases)			
	ii) Does he/she **incessantly** repeat things/T.V serial dialogue regardless of meaning/ context, whatever he/she has heard **later on**?	* Delayed echolalia			

	iii) ***For children aged 4 years or more**:* Does your child **usually** use "I for me" and "me for you" incorrectly?<br>*E.g., when you ask "do you want milk?" he/she says "yes, you want milk" or "Rohit wants milk"* (referring to him self).	* Pronoun reversal	◯		
	iv) ***For children aged 4 years or more**:* During conversation does your child **often** speak 'out of context' or irrelevantly?	Out-of-context speech and neologisms	◯		
	v) * ***For children aged 6 years or more:***<br>Does your child understand that somebody is making fun of him/her or can he/she understands jokes?	Child's response to an age-appropriate joke	◯		
	vi) Does your child keep on **repeating** any of the followings, like<br>• flapping hands,<br>• hand wringing,<br>• toe-walking,<br>• rocking or spinning,<br>• making **unusual** finger or hand movements near his/her face?	* Any type of motor stereotypes, unusual finger/hand movements near face	◯		
	vii) * Does your child have **inappropriate** fascination with movement?	Child's **inappropriate** fascination with objects in motion	◯		

	E.g. spinning wheels, opening and closing of doors, electric fan, running water and any other revolving object etc.				
Section A2b<br><br>Routines	Does your child unreasonably **insist** on doing things in a particular way and/or become **upset** if there is any change in the daily routine?<br><br>*E.g., Taking exactly the same route to the school or market, insisting on food being served in the same pattern or sequence etc.*	Child's insistence on any unusual routines or rituals	○		
Section A2c<br><br>Fixed interest	Does your child prefer to play with a particular **part** of a toy/object rather than the whole toy/object?<br>*E.g. wheels of a toy rather than the whole toy*<br><br>And/ Or<br>Persistent unusual preoccupation with inanimate objects? E.g. Toffee wrappers, threads, bits of papers, flowing water<br>And/ Or<br>Persistent behavioural attributes? E.g. Liking particular sound/ visual stimuli, any particular color or form of cloth	* Quality of child's play with different toys and objects	○		

Section A2d<br><br>Sensory symptoms	i) Is your child indifferent to pain or temperature?	Apparent indifference to pain or temperature	◯		
	ii) Does your child show excess reaction to specific sound or texture	Getting irritated with certain specific sounds or texture of certain clothes	◯		
	iii) Does your child have excessive smelling?	Excessive smelling of hands or arms	◯		
	iv) Does your child have excessive touching of objects?	Excessive touching objects in the room	◯		

SECTION B Complete this section (1-4) based on responses from section A

**1. No. of criteria fulfilled in A1 of the section A (Social Interaction and communication)**<br>**0:** Two or less<br>**1:** Three	☐
**2. No. of criteria fulfilled in A2 of the section A (restrictive and repetitive)**<br>**0:** Nil or one<br>**1:** Two or more	☐
**3. Is there onset at early development?**<br>**0:** No<br>**1:** Yes	☐
**4. Is there an impaired functioning?**<br>**0:** No<br>**1:** Yes	☐
**5. Interpretation of questionnaire (1 to 4)**<br>**0: No ASD** (If response to any of 1-4 is "0")<br>**1: ASD present** (If response to 1-4 is "1")	☐
**6. Total number of criteria fulfilled in A1 and A2 together**<br>**0. Four or less**<br>**1. Five or more**	☐
**7. Summary assessment of ASD**<br>**0: No ASD** (Response to 5 and 6 is "0")<br>**1: ASD** (Response to 5 and 6 is "1" and 8 is "0")	☐
**8. Can these symptoms be solely explained by Intellectual Disability?**<br>0: No 1: Yes<br>**Additional Notes if any:**	☐

Name of the Assessor	Signature of the Assessor	Date of assessment
